# Bevacizumab for Vestibular Schwannomas in Neurofibromatosis Type 2: A Systematic Review of Tumor Control and Hearing Preservation

**DOI:** 10.3390/jcm13237488

**Published:** 2024-12-09

**Authors:** Melina Screnci, Mathilde Puechmaille, Quentin Berton, Toufic Khalil, Thierry Mom, Guillaume Coll

**Affiliations:** 1 Département de Neurochirurgie, CHU Clermont-Ferrand, 63000 Clermont-Ferrand, France; mscrenci@chu-clermontferrand.fr (M.S.); qberton@chu-clermontferrand.fr (Q.B.); tkhalil@chu-clermontferrand.fr (T.K.); 2 Unité CRECHE, CIC 1405, INSERM, CHU Clermont-Ferrand, 63000 Clermont-Ferrand, France; tmom@chu-clermontferrand.fr; 3 Département d’Otorhinolaryngologie et de Chirurgie Cervico-Faciale, CHU Clermont-Ferrand, 63000 Clermont-Ferrand, France; mpuechmaille@chu-clermontferrand.fr; 4 Mixt Unit of Research (UMR) 1107, National Institute of Health and Medical Research (INSERM), University of Clermont Auvergne (UCA), 63000 Clermont-Ferrand, France

**Keywords:** vestibular schwannoma, bevacizumab, neurofibromatosis type 2, tumor volume reduction, hearing preservation, adverse effects

## Abstract

**Background/Objectives**: Vestibular schwannomas (VSs), also called acoustic neuromas, are benign tumors affecting the vestibulocochlear nerve, often leading to hearing loss and balance issues. This condition is particularly challenging in patients with neurofibromatosis type 2 (NF2), where VSs tend to develop bilaterally. Conventional treatments, such as surgery and radiotherapy, although effective, carry risks like hearing loss and nerve damage. Bevacizumab, a VEGF-targeting monoclonal antibody, has emerged as a less invasive treatment option, showing potential for tumor volume reduction and hearing preservation. This systematic review aims to assess the efficacy of bevacizumab in controlling tumor volume, preserving hearing, and identifying associated adverse events. **Methods**: A comprehensive systematic review was performed using PRISMA guidelines. PubMed and Cochrane Library databases were searched for studies evaluating the effects of bevacizumab on VS, focusing on key outcomes like tumor volume reduction, hearing preservation, and adverse events. Data extraction and quality assessment were independently conducted by two reviewers using the Newcastle-Ottawa Scale. **Results**: Nine studies involving 176 patients were included. Bevacizumab showed a partial tumor volume reduction (≥20%) in 40% of cases and disease stabilization in 50%, while 10% experienced tumor progression. Hearing outcomes revealed improvement in 36% of patients, stabilization in 46%, and deterioration in 18%. Severe adverse effects, including hypertension and thromboembolic events, occurred in 13% of patients, while 18% reported no side effects. Tumor regrowth was observed in some patients after treatment discontinuation, emphasizing the need for long-term monitoring. **Conclusions**: Bevacizumab demonstrates effectiveness in managing VS, particularly in NF2 patients, by reducing tumor size and preserving hearing in a substantial proportion of cases. However, the variability in patient response and the risk of adverse events underscore the need for individualized treatment approaches and further research, including randomized controlled trials, to optimize its clinical application.

## 1. Introduction

Vestibular schwannomas (VSs), commonly referred to as acoustic neuromas, are benign but potentially debilitating tumors that develop from Schwann cells on the vestibulocochlear nerve. In VS, these cells proliferate abnormally, resulting in tumors that often lead to hearing loss, balance disturbances, and, in severe cases, brainstem compression. This condition is particularly challenging in patients with neurofibromatosis type 2 (NF2), where VSs frequently present bilaterally, significantly complicating management. NF2 is an autosomal dominant genetic disorder caused by mutations in the NF2 gene located on chromosome 22, which encodes the protein merlin [1]. Merlin normally functions as a tumor suppressor, regulating Schwann cell growth and maintaining cellular shape and structure [1]. When NF2 gene mutations lead to the loss of merlin function, Schwann cells undergo uncontrolled proliferation, resulting in the formation of vestibular schwannomas and other tumors associated with NF2 [1].

This condition is particularly challenging in patients with neurofibromatosis type 2 (NF2), a rare autosomal dominant genetic disorder with an estimated birth incidence of 1 in 25,000 to 33,000 [1]. Unlike sporadic cases, NF2-associated vestibular schwannomas (VSs) often occur bilaterally and at a younger age, typically in adolescence or early adulthood, due to the genetic predisposition for tumor development. NF2 accounts for approximately 7% of all VS cases, a prevalence higher than previously estimated, likely due to improved identification of mosaic forms of the disease [2]. NF2-related tumors pose additional management challenges, as they tend to be more aggressive and are frequently accompanied by other tumors, such as meningiomas and spinal schwannomas, which further complicates the clinical picture [3]. Conventional treatment options, including surgery and radiotherapy, while effective in controlling tumor growth, carry high risks of hearing deterioration and cranial nerve damage. These risks are particularly significant in NF2 patients, whose outcomes are often less favorable compared to those with sporadic, unilateral VS [4]. In NF2 cases, the timing of interventions has historically been a topic of debate; however, research suggests that early proactive management may improve outcomes in preserving auditory and facial nerve function [3]. Given these risks and challenges, interest has grown in exploring less invasive treatments that emphasize both tumor volume control and hearing preservation. The need for strategies that allow for early detection and treatment is underscored by proactive screening programs, such as the Manchester NF2 Register, which emphasizes MRI monitoring in NF2-affected families [5]. This approach enables the identification of VS at smaller sizes, which can facilitate management and aid in preserving auditory function, a primary goal given the high likelihood of bilateral VS in NF2 patients [2]. As a result, there has been growing interest in exploring less invasive therapeutic options that prioritize both tumor volume control and hearing preservation.

Bevacizumab, a monoclonal antibody targeting vascular endothelial growth factor (VEGF), has emerged as a promising alternative for patients with VS, particularly those with NF2. This drug, initially developed for the treatment of cancers, has shown potential in slowing or halting tumor progression in VS, with early studies indicating promising results in both tumor volume reduction and hearing stabilization [6,7]. The primary therapeutic goals with bevacizumab involve achieving significant tumor shrinkage while simultaneously preserving or improving hearing function [8]. As tumor growth and progressive hearing loss are two of the most critical challenges in the management of VS, bevacizumab offers a non-surgical option that may reduce the need for more invasive interventions [9].

In addition to its primary objectives, the safety profile of bevacizumab has been extensively evaluated. While generally well tolerated, the drug is not without risks. Adverse events related to bevacizumab treatment, particularly severe toxicities such as Grade 3 and 4 events, including hypertension, proteinuria, and fatigue, have been reported in clinical studies [9,10]. Moreover, concerns have been raised regarding the potential for rapid tumor regrowth after discontinuation of the therapy, further emphasizing the need for careful long-term monitoring of patients undergoing treatment with bevacizumab [11].

The primary objective of this study is to conduct a systematic literature review, following the PRISMA guidelines, to evaluate the efficacy of bevacizumab in controlling tumor volume and preserving hearing function in patients with VS and NF2. Secondary outcomes include an assessment of adverse events associated with bevacizumab, with particular focus on severe toxicities and the risk of tumor rebound after treatment discontinuation. By clarifying the therapeutic benefits and potential risks, this review aims to guide the clinical use of bevacizumab in the management of VS.

## 2. Materials and Methods

### 2.1. Data Sources

Our systematic review adhered to the PRISMA 2020 guidelines Supplementary Materials. A comprehensive search was conducted in PubMed and the Cochrane Library databases on 15 August 2024, using the following keywords: “Neurinoma”, “Vestibular Schwannoma”, “Avastin”, and “Bevacizumab” in various combinations (Supplementary Materials). The search strategy was designed to capture all relevant studies investigating the effects of bevacizumab on VS, including studies only related to neurofibromatosis type 2. The protocol was not submitted to PROSPERO as it did not initially meet our institutional requirements for formal pre-registration; we instead prioritized the rigorous development of the methodology and eligibility criteria from the outset, ensuring transparency and methodological consistency.

### 2.2. Eligibility Criteria

Inclusion and exclusion criteria (Table 1) were defined prior to the commencement of the review. Our research question was structured using PICOS terms to ensure clear eligibility criteria.

1. Population: This review focused on men and women of all ages with unilateral or bilateral vestibular schwannomas (VSs), whether or not they were associated with neurofibromatosis type 2 (NF2). To maintain specificity, studies exclusively targeting pediatric populations, those involving other tumor types, or those examining schwannomas in non-vestibular locations were excluded. Importantly, studies that included both adult and pediatric populations were considered, allowing for a broader analysis across age groups. However, studies involving only pediatric patients were not included. Additionally, studies that did not address the defined clinical outcomes (e.g., tumor volume, hearing preservation, adverse effects) were excluded from this review.
2. Intervention: Only studies examining the use of bevacizumab as a treatment for VS were included, allowing us to assess its impact on effectiveness and tolerability specifically. Studies that assessed other treatments or alternative therapies were excluded to preserve a consistent analytical focus on bevacizumab.
3. Comparison: although a specific comparison group was not required, studies that included comparative data (e.g., placebo or alternative treatments) were not excluded if they otherwise met the inclusion criteria.
4. Outcomes: Key clinical outcomes were tumor volume reduction, hearing preservation, and adverse events. Studies were included only if they reported data on these outcomes. Articles with insufficient or irrelevant clinical data were excluded, as were those with fewer than five patients, to ensure the quality and robustness of the data included.
5. Study design: We included a range of study types, including observational studies, case series, and clinical trials, as these designs provided relevant data on bevacizumab’s impact on VS. Articles not published in English, those not freely accessible, or those missing key outcome data were excluded to maintain the review’s focus and relevance.

### 2.3. Main Outcome Measures

The primary objective of this review was to evaluate the efficacy of bevacizumab in controlling the tumor volume and preserving hearing function in patients with VS and NF2. Secondary outcomes included the assessment of adverse events related to the use of bevacizumab, particularly focusing on severe toxicities (Grade 3 and 4) and other side effects reported in the literature.

### 2.4. Data Collection and Analysis

Two independent reviewers (MS and GC) screened the titles, abstracts, and full texts of potentially relevant studies using a standardized data collection form. Discrepancies in the selection process were resolved through discussion, and in cases where a consensus could not be reached, a third reviewer was consulted to ensure the objectivity of the selection process. Data extraction focused on study characteristics, patient demographics, details of the vestibular schwannoma, treatment specifics, and clinical outcomes, which included tumor volume response, hearing outcomes, and adverse effects.

### 2.5. Data Extraction and Quality Assessment

The extraction of data was conducted independently by two reviewers using a standardized data collection form. The extracted data included study characteristics such as the author, year of publication, and study design, as well as patient demographics, tumor characteristics, details of the bevacizumab treatment, and relevant clinical outcomes. The quality of the included studies was assessed using the Newcastle–Ottawa Scale (NOS) for cohort studies, which evaluates the selection of study groups, comparability between groups, and the determination of the outcome. Disagreements between the reviewers were resolved by consensus, or by consulting a third reviewer if necessary.

### 2.6. Statistical Analysis

Statistical analyses were conducted using IBM Corp. Released 2022. IBM SPSS Statistics for Macintosh, version 29.0.0.0 (241). Armonk, NY, USA: IBM Corp. For the statistical analysis, weighted averages were calculated for each outcome category, including tumor volume response, hearing preservation, and adverse effects. The weighted averages took into account the differences in sample sizes across studies to provide a more accurate representation of the results. The formula used for calculating the weighted averages was as follows:

$$\mathrm{Weighted}\;\mathrm{Average}=\frac{\sum \left(x_{i}\times n_{i}\right)}{\sum n_{i}}$$

In this formula, $x_{i}$ represents the observed outcome (e.g., the percentage of partial tumor response) in study i, $n_{i}$ is the sample size of study *i*, and ∑ is the summation of contributions from all studies.

## 3. Results

**Figure 1:**
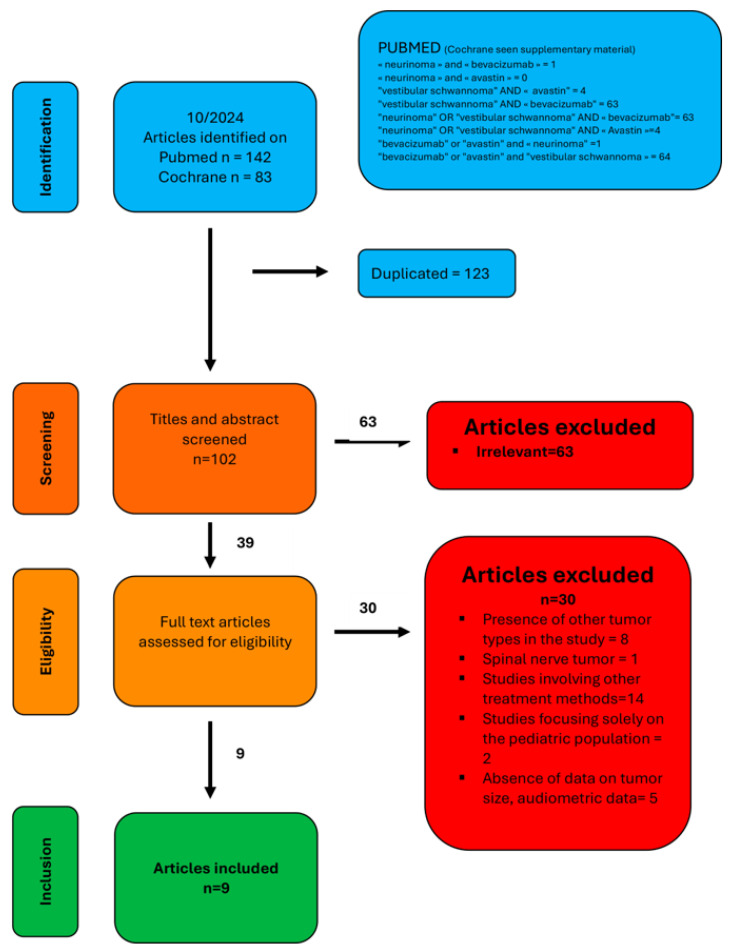
PRISMA flow diagram illustrating the systematic review process. Out of 225 identified articles, 39 met the inclusion criteria. After applying exclusion criteria, 9 articles were included in the final analysis.

After a comprehensive literature search (flowchart in Figure 1), nine relevant studies were identified and included in this systematic review [6,7,8,9,10,11,12,13]. These studies collectively involved a substantial patient cohort, providing diverse data on the effects of bevacizumab in the treatment of VS with NF2. The studies varied in their methodologies, patient populations, and reported outcomes, but all contributed valuable insights into the effectiveness and risks associated with bevacizumab, particularly concerning tumor volume control, hearing preservation, and adverse effects.

The characteristics of the included studies are detailed in Table 2.

### 3.1. Quality Assessment of Included Studies (Table 3)

The quality of the studies included in this review was assessed using the Newcastle–Ottawa Scale (NOS). The results of the assessment revealed that most studies were of high quality, with scores ranging from 7 to 9 out of a possible 9 points. Notably, four studies [6,7,12,13] achieved the highest scores, each receiving a total of 9 points. These studies demonstrated strong methodological designs, characterized by well-defined selection criteria, robust comparability of cohorts, and comprehensive outcome assessments, indicating a solid foundation for the reported findings. Three studies [9,10,14] received 8 points, reflecting high-quality research with clear selection processes and outcome measures, though some limitations in comparability were noted. The two remaining studies [8,11] received 7 points, indicating well-conducted studies but with room for improvement in the outcome reporting or selection processes.

**Table 3:** Quality assessment of included studies using the Newcastle–Ottawa Scale.

Study	Selection	Comparability	Outcome	Total Score
Blakeley [13]	4	2	3	9
Douwes [12]	4	2	3	9
Fuji [8]	3	2	2	7
Gugel [10]	4	2	2	8
Killeen [9]	3	2	3	8
Morris [6]	4	2	3	9
Plotkin [7]	4	2	3	9
Webb [11]	3	2	2	7
Sverak [14]	4	2	2	8

### 3.2. Patient Demographics (Table 2)

A total of 176 NF2 patients were included in this analysis. The weighted average age across the studies for patients treated with bevacizumab was 27.04 years. The weighted age range was between 12.35 years and 58.06 years. The male-to-female ratio was 86/90.

### 3.3. Tumor Volume Control (Table 4)

The studies indicated that bevacizumab was associated with a significant reduction in tumor volume in a substantial proportion of patients. Rates of partial response (significant reduction in tumor volume) ranged from 31% to 64% across studies, with a weighted average of 40%. Additionally, stable disease (no tumor progression) was observed in 50% of patients, while only 10% experienced tumor progression despite treatment.

**Table 4:** Summary of results with weighted averages.

Outcome	Weighted Average
Partial tumor response	40%
Stable disease	50%
Progressive disease	10%
Hearing improvement	36%
Stable hearing	46%
Hearing Loss	18%
Severe adverse effects (Grade 3/4)	13%
Other adverse effects	69%
No adverse effects	18%

### 3.4. Hearing Preservation (Table 4)

Regarding the effect of bevacizumab on hearing function, the results showed hearing improvement in 36% of patients, 46% maintained stable hearing during treatment, while 18% experienced hearing loss despite bevacizumab therapy. These findings suggest that bevacizumab is generally effective in stabilizing hearing function in patients with VS, with moderate rates of improvement.

### 3.5. Adverse Effects (Table 4)

Adverse effects associated with bevacizumab administration were reported in all studies, although their severity and frequency varied. Severe adverse effects (Grade 3 or 4), such as severe hypertension or thromboembolic complications, were reported in 13% of patients. Other, less severe side effects, including fatigue, headaches, and gastrointestinal symptoms, were observed in 69% of patients. There was no adverse effect in 18% of cases. No treatment-related deaths were reported in any of the included studies.

## 4. Discussion

The findings from this systematic review, which pooled data from nine studies, offer a comprehensive overview of the potential benefits and limitations of bevacizumab as a treatment strategy in the management of VS, particularly in patients with NF2. This review included a mixed population of both adult and pediatric patients, allowing for a broader scope of findings; this approach is particularly valuable as mixed populations can better capture the range of tumor responses and toxicities associated with bevacizumab.

Excluding studies focused exclusively on pediatric populations is justified by the significant differences in pharmacodynamics and treatment responses observed between pediatric and adult patients. Research indicates that bevacizumab’s efficacy and safety profile can vary across age groups due to differences in tumor biology, growth rates, and metabolism. Pediatric patients with NF2 often present more aggressive VS growth patterns and may experience different treatment outcomes, as seen in studies where tumor responses and hearing improvements were notably lower in children compared to adults [15,16]. Additionally, children may require extended or alternative treatment regimens, as responses in pediatric populations are often transient and may relapse once therapy is discontinued [16].

By prioritizing studies with mixed-age cohorts or adult populations, this review aims to maintain a more consistent analytical basis for evaluating bevacizumab’s effects on tumor volume, hearing preservation, and adverse events in NF2-related VS. While this approach may not capture the full spectrum of responses in exclusively pediatric cases, it could help to reduce variability and may improve the relevance of findings to the broader NF2 patient population, particularly for applications in standard adult clinical practice.

The results of our analysis indicate that bevacizumab is associated with significant tumor volume reduction in a considerable proportion of patients and contributes to hearing preservation in a substantial number of cases. However, the treatment is not without risks, as adverse effects, including severe toxicities, were reported across multiple studies.

### 4.1. Methodology Discussion

The studies included in this review were evaluated using the NOS, with most scoring high, reflecting robust methodologies. Blakeley et al. [13], Plotkin et al. [7], Morris et al. [6] and Douwes [12], scoring 9 points, had well-defined cohorts and comprehensive data on tumor control, hearing preservation, and adverse events. These high scores reinforce confidence in their findings. In contrast, Fujii et al. [8] and Webb et al. [11] scored lower, at 7 points each, due to issues with baseline comparability and sample size discrepancies. These limitations raise concerns about potential bias in their results. Gugel et al. [10], Killeen et al. [9] and Sverak et al. [14], both scoring 8 points, exhibited solid methodologies but were limited by smaller sample sizes and shorter follow-ups. Overall, the NOS assessment suggests that the studies included in this review provide a reliable basis for evaluating the effectiveness and safety of bevacizumab in the treatment of VS.

### 4.2. Tumor Volume Control

One of the primary goals of treating VS with bevacizumab is to reduce tumor volume, particularly in patients with progressive or symptomatic disease. On average, our literature review indicates that 40% of patients showed a partial tumor response, defined as a reduction in tumor volume of at least 20% [6,7,8]. This finding aligns with adult-focused NF2 studies, which report similar outcomes in tumor volume reduction. For instance, Fujii et al. [8] observed a partial tumor response in 53% of patients, while Douwes et al. [12] reported a 31% response rate. This variation highlights that the efficacy of bevacizumab may differ between studies, though it remains consistent with our overall findings.

In pediatric studies, however, partial tumor response rates are generally lower. For example, Hochart et al. (2015) reported minimal volumetric response in a pediatric cohort, observing only one major and two minor responses among seven children treated for NF2-related vestibular schwannomas, with tumor stabilization being more commonly achieved [15]. Similarly, Renzi et al. found only two cases of tumor shrinkage out of seventeen pediatric patients, further emphasizing that while bevacizumab may slow tumor progression in younger populations, significant reductions in volume are less frequent [16].

Tumor stabilization, meaning no further progression, was observed in 50% of patients in our analysis, indicating a meaningful role for bevacizumab in controlling tumor growth. Douwes et al. [12] reported stabilization in 69% of their patients, and Morris et al. [6] found 51% stabilization. These findings confirm bevacizumab’s significant impact in slowing tumor growth, which is particularly beneficial for delaying more invasive interventions such as surgery or radiotherapy. In cases of NF2, where tumor growth can lead to serious complications such as brainstem compression and cranial nerve dysfunction, stabilizing tumor growth becomes a crucial therapeutic objective [12,13].

Pediatric studies support this benefit of stabilization: while significant shrinkage may be limited, bevacizumab frequently slows growth rates, as shown by Hochart et al., who observed a median annual tumor growth reduction from 138% to 36% after one year of treatment [15].

However, around 10% of patients exhibited disease progression despite bevacizumab treatment, underscoring the potential limitations of bevacizumab in controlling particularly aggressive or resistant tumor phenotypes, which may necessitate alternative strategies or continued treatment in some cases [11]. Notably, similar findings are seen in pediatric cohorts; Renzi et al. reported cases of tumor progression and worsening hearing loss upon cessation of treatment, emphasizing that the therapeutic effects may be less durable in younger populations, potentially requiring longer or maintenance therapy to sustain benefits [16].

Webb et al. reported cases of tumor regrowth in 26% of patients after discontinuing bevacizumab, highlighting the need for ongoing monitoring after treatment cessation [11].

This ongoing monitoring is particularly crucial in pediatric cases, where treatment cessation has been associated with high rates of recurrence. The variability in tumor response rates across age groups and studies suggests the importance of tailored treatment approaches and further investigation into predictive biomarkers that could better inform therapeutic decisions in NF2 patients, particularly among younger patients where the long-term efficacy and safety of bevacizumab remain areas of active research

### 4.3. Hearing Preservation

Preserving auditory function is a major therapeutic goal in managing VS, especially since traditional treatments like surgery often result in further hearing loss. Our data show that 36% of patients experienced hearing improvement during bevacizumab treatment, and 46% maintained stable hearing. In comparison, Douwes et al. [12] reported hearing improvement in 40% of patients and stabilization in 53%, while Plotkin et al. [7] noted improvement in 41%. These figures are slightly higher than the overall results, suggesting notable effectiveness in preserving hearing.

However, in pediatric populations, hearing preservation results tend to vary. Renzi et al. found that 61% of children and adolescents with NF2 experienced hearing improvement within six months of bevacizumab treatment, which aligns well with adult outcomes [16]. However, most pediatric cases saw hearing loss resume upon treatment cessation, indicating a potentially lower durability of hearing benefits in younger patients. Similarly, Hochart et al. observed limited hearing improvement in their cohort, with only one child achieving a significant auditory response over the course of treatment [15].

The ability of bevacizumab to stabilize auditory function is particularly important for NF2 patients, where bilateral tumors can lead to significant hearing loss, severely impacting quality of life. Maintaining hearing can delay the need for interventions like cochlear implants or hearing aids, which often become less effective as tumor progression worsens [6,7].

This is especially relevant in pediatric cases, as early hearing preservation can mitigate the social and developmental impacts of hearing loss during childhood and adolescence [16].

Nevertheless, 18% of patients experienced hearing loss during treatment, indicating that bevacizumab does not universally protect auditory function. In pediatric studies, such as those by Renzi et al., the rates of hearing deterioration were similarly significant upon treatment discontinuation [16]. This demonstrates the complexity of the interactions between tumor size reduction and hearing preservation, especially in cases of residual tumors following partial resection. Additionally, it highlights the importance of future research to identify patient characteristics that may predict auditory outcomes and guide therapeutic strategies, as the responses to bevacizumab appear to vary significantly between age groups [15].

### 4.4. Adverse Effects

While bevacizumab offers notable benefits in tumor control and hearing preservation, its use is associated with a range of adverse effects. Approximately 69% of patients reported side effects, most of which were mild to moderate in intensity [9,10]. Common side effects include fatigue, headaches, and gastrointestinal discomfort, which, though generally manageable, may affect the quality of life in some patients.

Serious adverse events, classified as grade 3 or 4 toxicities, were observed in 13% of patients. The most frequent severe side effects include hypertension, proteinuria, and thromboembolic events [9]. For example, Morris et al. [6] reported cases of hypertension in 30% of patients, and Blakeley et al. [13] emphasized the need to monitor cardiovascular effects throughout the duration of treatment. Plotkin et al. further highlighted that, while most adverse events were mild, a subset of patients experienced significant liver enzyme elevations, proteinuria, and hypertension, underscoring the cumulative risk associated with chronic bevacizumab therapy in NF2 patients who may require extended treatment durations [17]. In pediatric populations, adverse effects of bevacizumab appear more pronounced. Spini et al. demonstrated a high prevalence of serious adverse events in pediatric patients with solid tumors undergoing anti-angiogenic therapy, including bevacizumab [18]. In their comprehensive meta-analysis, they found that 46% of pediatric patients experienced severe adverse effects, with cardiovascular complications and hematologic toxicities being particularly common. Similarly, Hochart et al. reported that two of their seven pediatric patients experienced severe adverse effects, leading to treatment discontinuation in one case due to osteomyelitis, underscoring the unique vulnerability of pediatric patients to intensive anti-angiogenic therapy [16]. Renzi et al. also noted that although bevacizumab was well-tolerated in most cases, continuous monitoring remains critical due to occasional but severe adverse events among children and adolescents [16]. These findings underscore the need for stringent monitoring protocols, especially in vulnerable populations such as pediatric and NF2 patients, to address both immediate and long-term adverse effects and improve treatment safety. Regular cardiovascular assessments and active hypertension management are essential components of long-term therapy with bevacizumab, as recommended in several studies [5,8].

Plotkin et al. [7] and Blakeley et al. [13] further stressed the importance of monitoring patients for signs of cardiovascular complications, particularly given the chronic nature of bevacizumab therapy in NF2 patients. These studies recommend regular cardiovascular assessments and management of hypertension as part of the treatment protocol to minimize the risks of long-term therapy.

### 4.5. Clinical Implications and Future Directions

The evidence gathered from these studies highlights the need for more personalized treatment approaches in VS. The variability in tumor responses and hearing outcomes suggests that not all patients benefit equally from bevacizumab. Identifying biomarkers that predict responsiveness to bevacizumab could help tailor treatments to those most likely to benefit, minimizing unnecessary exposure to the drug’s adverse effects.

Additionally, there is a need for randomized controlled trials (RCTs) to better define bevacizumab’s efficacy compared to other treatment options, such as surgery or radiation therapy. Most of the current evidence is based on retrospective studies or single-arm trials, which limits the ability to draw definitive conclusions about its comparative effectiveness. RCTs would help establish standardized dosing regimens and define the optimal timing for treatment initiation and discontinuation.

Furthermore, long-term studies are necessary to assess the durability of tumor responses and hearing preservation, particularly in NF2 patients who often require lifelong management. Ongoing research should also focus on strategies to manage the risks of rebound tumor growth post-treatment, as highlighted by Webb et al. [11], and to refine follow-up protocols to catch early signs of recurrence.

### 4.6. Bias and Limitations

The retrospective nature of many studies, such as those by Fujii et al. [8] and Webb et al. [11], may impact the quality of the data due to reliance on historical records and the lack of prospective control, potentially leading to an overestimation of bevacizumab’s effectiveness. Additionally, publication bias may favor positive results, while negative outcomes might be underreported. Differences in study designs, follow-up periods, and outcome definitions may complicate cross-study comparisons. The lack of randomized controlled trials is a significant limitation, as randomization would help clarify bevacizumab’s efficacy relative to other treatment options like surgery or radiotherapy.

## 5. Conclusions

VSs, especially in patients with NF2, pose a significant challenge due to tumor growth and its impact on hearing and nerve function. While surgery and radiotherapy are effective, they carry risks of hearing loss and nerve damage. Bevacizumab, a VEGF-targeting monoclonal antibody, has emerged as a promising alternative for tumor control and hearing preservation. This review of nine studies shows that bevacizumab leads to partial tumor shrinkage in 40% of patients and disease stabilization in 50%, making it especially useful for NF2 patients. Additionally, hearing preservation was achieved in 46% of cases, with 36% experiencing improvement. However, adverse effects, including severe toxicities, were reported in 13% of patients, highlighting the need for careful monitoring. Despite its benefits, variability in patient responses and risks like rebound tumor growth after treatment discontinuation call for further research. Future studies should focus on predicting patient response and optimizing treatment. Randomized controlled trials will help solidify bevacizumab’s role compared to traditional therapies.

## Figures and Tables

**Table 1:** Inclusion and exclusion criteria for study selection.

Inclusion Criteria	Exclusion Criteria
Studies on vestibular schwannoma	Presence of other tumor types in the study (e.g., meningioma)
Use of bevacizumab as a treatment	Presence of other tumor localizations (e.g., spinal nerve tumors)
Availability of data on tumor size	Studies involving other treatment methods
Availability of audiometric data	Studies focusing solely on the pediatric population
Availability of data on side effects	Absence of data on tumor size, audiometric data
Articles published in English	Articles not published in English

**Table 2:** Results from studies on bevacizumab treatment for VS in patients with NF2: tumor response, hearing outcomes, and complications.

Studies	Tumor Volume Response (*n* = Number of Tumors)				Hearing Outcome (*n* = Number of Ear)			Adverse Event (*n* = Number of Patients)			Age	Sex (M/F)
	Complete	Partial	Stable	Progressive	Improvement	Stable	Loss	Grade 3 o 4	Other	None		
Morris 2016 [6]	0/61 (0%)	24/61 (39%)	28/61 (46%)	9/61 (15%)	3/33 (9%)	28/33 (85%)	2/33 (6%)	8/61 (13%)	42/61 (68%)	11/61 (18%)	25 (10–57)	36/25
Fuji 2020 [8]	0/15 (0%)	8/15 (53%)	5/15 (33%)	2/15 (13%)	1/4 (25%)	2/4 (50%)	1/4 (25%)	0/10 (0%)	5/10 (50%)	5/10 (50%)	30.5 (19.5–38.75)	1/9
Douwes 2024 [12]	0/16 (0%)	5/16 (31%)	11/16 (69%)	0/16 (0%)	6/15 (40%)	8/15 (53%)	1/15 (7%)	2/17 (12%)	14/17 (82%)	1/17 (6%)	48.1 (22.9–70.7)	8/9
Blakeley 2026 [13]	0/28 (0%)	18/28 (64%)	10/28 (36%)	0/28 (0%)	9/19 (47%)	-	-	3/14 (21%)	-	-	30.5 (14–79)	4/10
Killeen 2019 [9]	-	-	-	-	0/12 (0%)	8/12 (67%)	4/12 (33%)	-	-	-	17 (12–47)	4/3
Gugel 2019 [10]	0/16 (0%)	18/28 (64%)	11/16 (69%)	4/16 (25%)	8/12 (67%)	3/12 (25%)	1/12 (8%)	2/9 (22%)	2/9 (22%)	5/9 (56%)	21 (19–25)	2/7
Plotkin 2019 [7]	0/41 (0%)	15/41 (37%)	24/41 (58%)	2/41 (5%)	9/21 (43%)	2/21 (9%)	10/21 (48%)	3/22 (14%)	20/22 (91%)	0/22 (0%)	23 (12–62)	9/13
Webb 2023 [11]	0/16 (0%)	5/16 (31%)	9/16 (56%)	2/16 (13%)	5/9 (56%)	1/9 (11%)	3/9 (33%)	-	-	-	19 (0.5–61)	12/7
Sverak 2019 [14]	0/17 (0%)	8/17 (47%)	8/17 (47%)	1/17 (6%)	5/9 (56%)	2/9 (22%)	2/9 (22%)	-	-	-	30 (15–57)	10/7

## Data Availability

The data are not stored as this study is a systematic review conducted according to the PRISMA guidelines, and the data used are publicly accessible in the original studies referenced.

## Supplementary Materials

The following supporting information can be downloaded at: https://www.mdpi.com/article/10.3390/jcm13237488/s1, Search string and PRISMA Checklist.
