# Supplementary material for: Bevacizumab for Vestibular Schwannomas in Neurofibromatosis Type 2: A Systematic Review of Tumor Control and Hearing Preservation
Source: J Clin Med. 2024 Dec 9;13(23):7488. doi: 10.3390/jcm13237488 (PMC11642482; doi:10.3390/jcm13237488)
Supplement: Supplementary file 1 [file jcm-13-07488-s001.zip › jcm-3266498-supplementary.pdf]

Below are the search strings used for each database:

PubMed:

- “neurinoma” AND “bevacizumab” = 1
- “neurinoma” AND “avastin” = 0
- “vestibular schwannoma” AND “avastin” = 4
- “vestibular schwannoma” AND “bevacizumab” = 63
- “neurinoma” OR “vestibular schwannoma” AND “bevacizumab” = 63
- “neurinoma” OR “vestibular schwannoma” AND “avastin” = 4
- “bevacizumab” OR “avastin” AND “neurinoma” = 1
- “bevacizumab” OR “avastin” AND “vestibular schwannoma” = 64

Cochrane Library:

- “neurinoma” AND “bevacizumab” = 0
- “neurinoma” AND “avastin” = 0
- “vestibular schwannoma” AND “avastin” = 1
- “vestibular schwannoma” AND “bevacizumab” = 4
- “neurinoma” OR “vestibular schwannoma” AND “bevacizumab” = 1
- “neurinoma” OR “vestibular schwannoma” AND “avastin” = 1
- “bevacizumab” OR “avastin” AND “neurinoma” = 36
- “bevacizumab” OR “avastin” AND “vestibular schwannoma” = 36

PRISMA 2020 Checklist

Section and Topic	Item #	Checklist item	Location where item is reported
TITLE	1	Identify the report as a systematic review.	Page 1 (Title)
ABSTRACT	2	Provide a structured summary compliant with PRISMA guidelines.	Page 1 (Abstract)
INTRODUCTION	3	Describe the rationale for the review in the context of existing knowledge.	Pages 1-2 (Introduction)
INTRODUCTION	4	Provide an explicit statement of the objectives or questions the review addresses.	Page 2 (Introduction)
METHODS	5	Specify the inclusion and exclusion criteria for the review, including populations, interventions, comparators, outcomes.	Page 3 (Methods)
METHODS	6	Identify all databases and sources searched for studies.	Page 3 (Methods)
METHODS	7	Present the detailed search strategies for all databases.	Page 3 (Methods)

METHODS	8	Describe methods used to decide study eligibility.	Page 4 (Methods)
METHODS	9	Describe data extraction methods from reports.	Page 4 (Methods)
METHODS	11	Describe tools used to assess risk of bias in included studies.	Page 4 (Methods)
METHODS	13a	Describe processes for determining study eligibility for synthesis.	Page 4 (Methods)
RESULTS	16a	Describe selection results, ideally with a flow diagram.	Page 5 (Results)
RESULTS	17	Cite each included study and present its characteristics.	Page 5 (Results, Table 1)
RESULTS	19	Present summary statistics and effect estimates for each study.	Page 6 (Results)
RESULTS	20a	Summarize characteristics and risk of bias of contributing studies.	Page 6 (Discussion)
DISCUSSION	23a	Provide a general interpretation of the results in the context of other evidence.	Pages 6-7 (Discussion)
DISCUSSION	23b	Discuss limitations of the evidence included and the review process.	Pages 8,11 (Discussion)

DISCUSSION	23d	Discuss implications of results for clinical practice and future research.	Pages 8-9 (Discussion)
OTHER INFORMATION	25	Describe sources of financial or non-financial support for the review.	Page 11-12
